# Overview of Cytomegalovirus Ocular Diseases: Retinitis, Corneal Endotheliitis, and Iridocyclitis

**DOI:** 10.3390/v16071110

**Published:** 2024-07-11

**Authors:** Reiko Kobayashi, Noriyasu Hashida

**Affiliations:** Department of Ophthalmology, Graduate School of Medicine, Osaka University, Osaka 565-0871, Japan

**Keywords:** cytomegalovirus infection, corneal endotheliitis, iridocyclitis, retinal uveitis

## Abstract

Cytomegalovirus (CMV) infection is a significant clinical concern in newborns, immunocompromised patients with acquired immunodeficiency syndrome (AIDS), and patients undergoing immunosuppressive therapy or chemotherapy. CMV infection affects many organs, such as the lungs, digestive organs, the central nerve system, and eyes. In addition, CMV infection sometimes occurs in immunocompetent individuals. CMV ocular diseases includes retinitis, corneal endotheliitis, and iridocyclitis. CMV retinitis often develops in infected newborns and immunocompromised patients. CMV corneal endotheliitis and iridocyclitis sometimes develop in immunocompetent individuals. Systemic infections and CMV ocular diseases often require systemic treatment in addition to topical treatment.

## 1. Introduction

Human cytomegalovirus (CMV) is a virus belonging to the β subfamily of the Herpesviridae family and has linear double-stranded DNA [1]. Histological findings have revealed inclusion bodies called “owl’s eye cells” in CMV-infected tissue specimens [2]. CMV infections can be divided into those found in newborns and immunocompetent hosts as well as those found in immunocompromised hosts [3]. Most infections occur in childhood, usually show no obvious pathogenicity, and are generally asymptomatic in immunocompetent hosts [1]. CMV is one of the major infectious agents causing congenital infection and causes thrombocytopenia, hemolytic anemia, pneumonia, gastrointestinal liver damage, and central nervous system damage in infected newborns [3,4,5,6,7]. In some neonates, the disease is severe and can lead to neonatal death in a small percentage of cases [6,7,8,9,10]. CMV infection also causes severe disease in immunocompromised individuals. For example, CMV infection is a significant clinical concern in solid organ and stem cell transplant recipients, patients receiving immunosuppressive therapy, (e.g., cancer patients receiving chemotherapy), and patients with acquired immunodeficiency syndrome (AIDS) due to human immunodeficiency virus (HIV) [11]. CMV can establish a lifelong latent infection after primary infection and periodically reactivates from a latent state [12,13,14]. The virus can become latent in mononuclear lymphocytes and bone marrow cells of immunocompetent hosts and reactivate under immunosuppressive conditions [1,15]. In immunocompromised hosts, reactivation of or re-infection with latently infected CMV is known to cause severe damage to the lungs, gastrointestinal tract, brain, spinal cord, and eyes [16,17]. Recently, it has also been shown that CMV infection causes some ocular diseases in immunocompetent individuals [18,19,20].

This article focuses on CMV-associated ocular diseases and describes their characteristics and treatment.

## 2. The Mechanism of CMV Infection

CMV can replicate in various cell types, including epithelial cells, endothelial cells, fibroblasts, myeloid-derived cells, and smooth muscle cells, facilitating viral spread within the host and infection between hosts [21,22,23]. Like many herpes viruses, CMV modulates host cells through interfering with essential signaling pathways for innate or acquired immune responses [3]. In addition, α herpesviruses such as human herpesvirus (HHV) latently infect neurons and can infect various host species. On the other hand, β herpesviruses such as CMV tend to be latent in lymphocytes of hematopoietic origin and exhibit host species specificity [24]. Once primary infection occurs, CMV spreads via the blood to various organs, becoming latent in circulating monocytes and bone marrow cells [25]. CMV-infected classical inflammatory monocytes recirculate into the bone marrow, carry the virus, and differentiate into patrolling monocytes with a long half-life. Once the monocytes are activated and differentiate into macrophages, viral replication may reinitiate [26].

Macrophages and their progenitor cells (monocytes and bone marrow cells) highly express pattern recognition receptors (PRRs) [27], which can detect common non-self pathogen-associated molecular patterns (PAMPs) and play an essential role in systemic CMV dissemination and latency [25,28,29,30,31]. B-cells and T-cells also play important roles in CMV infection. In particular, CD4+ and CD8+ T-cells control the balance between persistent and latent CMV infection throughout life [3,32,33,34].

Innate and acquired immune cells secrete cytokines and chemokines to organize the immune response. The interaction of the receptor with the pathogen initiates a signaling cascade that results in the transcriptional stimulation of numerous downstream cytokines. Cytokine signaling is a critical regulatory switch between initiating an immune response and maintaining peripheral homeostasis. Many cytokines function in an autocrine or paracrine positive feedback capacity [24]. Cytokines such as interleukins (ILs), interferons (IFNs), and hematopoietic growth factors activate the Janus kinase-signal transducer and activator of transcription (JAK-STAT) pathway [35,36]. Since homeostasis is most important for biological systems, the signaling also induces negative feedback factors such as suppressor of cytokine signaling (SOCS) proteins that help prevent adverse immunopathology [24]. SOCS proteins play an essential role in the cytokine signaling pathway [24]. SOCS proteins negatively regulate signaling pathways induced by antiviral and inflammatory cytokines [36,37]. Monocytes [38], macrophages [39], dendritic cells [40,41], microglia [42], neutrophils [43], NK cells [44], CD4+ and CD8+ T-cells [45,46], and Müller cells [47], among others, express SOCS proteins. Knockout mice models have verified the importance of SOCS1 and SOCS3 in regulating immune responses [24]. IFN signaling induces SOCS1 [48] which, in turn, suppresses Th1 lineage differentiation in CD4+ T-cells through regulating JAK-STAT pathway proteins [49,50]. SOCS1 can also modulate other cell signaling pathways, such as Toll-like receptor (TLR) signaling and macrophage activation [51]. On the other hand, the primary function of SOCS3 is to inhibit IL-6 family cytokine signaling by targeting the gp130 receptor [52,53]. In addition, SOCS3 can inhibit the development of CD4+ Th1 cells [54] and promote differentiation into the Th2 lineage [24]. In an experimental mouse model of AIDS-related CMV retinitis (experimental murine AIDS (MAIDS)-related mouse CMV (MCMV) retinitis), SOCS1 and SOCS3 were found to be involved in signal transduction during viral infection and were related to the severity of experimental MAIDS-related MCMV retinitis [24] (see Section 6. CMV Retinitis).

The extensive cell tropism of CMV requires the cooperative interaction of envelope glycoproteins and cell surface receptors [55]. The envelope of CMV contains many types of glycoproteins involved in viral adsorption, entry, and cell fusion, some of which are involved in determining the cell tropism [56].

Notably, gB (UL55), gN (UL73), gO (UL74), gH (UL75), gM (UL100), gL (UL115), gpUL128, gpUL130, and gpUL131A are CMV envelope glycoproteins, while heparan sulfate proteoglycans (HSPG), epidermal growth factor receptor (EGFR), platelet-derived growth factor α receptor (PDGFα), and integrins are their cellular receptors [57]. The main envelope glycoproteins tethering CMV to HSPGs are gB and gM/gN complexes [58,59]. The gB and gM/gN complexes mediate interactions between CMV and cell surface HSPGs, and they play an important role during attachment to the host cells [59,60]. CMV entry is dependent on β1 and β3 integrins [57,61]. Specifically, αVβ3 integrin has been reported to function as a gH-dependent co-receptor [62]. gB contains a disintegrin-like motif that binds to integrins [57,61,63]. In addition, α1β1, α2β1, α3β1, and α6β1 integrins have also been reported to play roles in the signaling pathway at the post-attachment step of CMV entry [58,61,64,65]. Moreover, gB is essential for cell-to-cell spread [55,58,66,67], and gN is involved in attachment of the virus to host cells and its spread [59,68,69]. As the gene encoding gN has a highly variable region, gN polymorphisms may allow the virus to evade neutralizing antibodies, promoting re-infection with CMV in seropositive individuals [70].

TLRs are the essential PRRs in CMV infection [71]. In the classical TLR2 pathway, when PAMP binds to the receptor, it activates the nuclear factor kappa-light-chain-enhancer of activated B cells (NF-κB) and mitogen-activated Protein (MAP) kinase pathways. It stimulates the transcription of various inflammatory cytokines, such as tumor necrosis factor-α (TNF-α), IL-6, and IFN-β [72,73]. The TLR2/TLR1 heterodimer complex serves as a crucial sensor for CMV [74]. gB and gH interact with TLR2 and TLR1. Integrins act as receptors for gB and gH, potentially enhancing the interaction of CMV with the TLR2/TLR1 heterodimer upon binding [74]. In addition, gB and gH interact directly with TLR2 on the plasma membrane, stimulating the NF-κB pathway and producing inflammatory cytokines [73,75]. Among microRNAs in CMV, miR-UL112-3p targets TLR2 and inhibits NF-κB signaling through TLR2 [73].

CMV adsorption and entry into fibroblasts require the trimer complex (TC) gH/gL/gO [58,76,77,78]. PDGFα functions as a cellular receptor for TC. Following the interaction of TC and PDGFα, the TC activates gB to directly fuse the viral envelope to the plasma membrane in a pH-independent manner [76,79]. In addition, the adsorption and entry of CMV into vascular endothelial, epithelial, and bone marrow cells require the pentameric complex (PC) gH/gL/gp (UL128, 130, and 131A) [76,77]. Neuropilin 2 (Nrp2) functions as a receptor for the PC. The interaction between PC and Nrp2 promotes the endocytosis of viral particles and activation of gB by the gH/gL complex, resulting in pH-dependent entry into the cell [76,80]. The cell surface protein CD147 is another CMV entry mediator. CD147 functions to facilitate CMV entry into epithelial and endothelial cells using the PC [81].

As a host-side mechanism, the cytoplasmic double-stranded DNA sensor Z-DNA-binding protein 1 activates CMV-mediated IFN regulatory factor 3 and suppresses CMV replication [82]. Additionally, IFN gamma-inducible protein 16 has been reported to inhibit CMV replication by directly suppressing specificity protein 1-mediated transcription of the CMV genes UL54 and UL44, which are crucial for viral DNA synthesis [83]. Furthermore, nucleotide-binding oligomerization domain 2, expressed on monocytes, macrophages, dendritic cells, and epithelial cells [84,85], has a role in reducing CMV replication and enhancing antiviral and pro-inflammatory cytokine responses [71].

## 3. CMV Infection in Immunocompetent Hosts

CMV persistently infects approximately 80% of the world’s population but does not usually cause disease in immunocompetent individuals [3,14]. Most CMV infections in immunocompetent individuals are asymptomatic, but primary infection can cause mononucleosis-like syndrome [86]. The clinical findings are similar to those for the primary infection, with fever, malaise, myalgia, headache, and fatigue being the most common signs and symptoms of this disease. A few patients may present with splenomegaly, hepatomegaly, lymphadenopathy, and rash [87,88]. CMV infection in immunologically immunocompetent individuals is usually considered less severe than the morbidity and mortality seen in immunocompromised individuals. Severe complications such as pneumonia, retinitis, hepatitis, encephalitis, and disseminated CMV disease with multi-organ damage are infrequent in immunologically healthy individuals [11,89]. CMV retinitis rarely occurs in immunocompetent individuals; however, these patients usually have some degree of immune dysfunction, such as advanced age, diabetes, corticosteroid use, or non-cytotoxic immunosuppressive drug use [90]. Conversely, CMV has been shown to cause iridocyclitis and corneal endotheliitis in immunocompetent individuals (discussed in detail in Section 7. CMV Corneal Endotheliitis and Section 8. CMV Iridocyclitis).

## 4. Congenital CMV Infection

CMV is a significant cause of congenital infection worldwide [4,6]. The prevalence of congenital CMV infection ranges from 0.2 to 2.0% of pregnancies (average 0.64%) [7]. Congenital CMV infection occurs when the virus crosses the placenta and is transmitted from the mother to the baby [7]. The majority (90%) of infants with congenital CMV infection are asymptomatic at birth. Of those infants who are symptomatic, 30–40% suffer from severe symptoms associated with congenital CMV infection [4,91,92]. Congenitally infected infants can suffer from multi-symptomatic diseases affecting many organ systems, such as pneumonia, gastrointestinal disease, retinal disease, and central nervous system (CNS) disease [4,5,9]. CNS abnormalities often cause a variety of neurological symptoms, including mental retardation, decreased motor skills, sensorineural hearing loss, and vision loss [3,4,7,10]. In addition, congenital CMV infection can cause jaundice, hepatitis, hepatosplenomegaly, petechiae, and thrombocytopenia in infected newborns [4,5] and, in a few cases, may lead to neonatal death [7,8,9,10]. About 20% of children with symptomatic congenital CMV infection have visual impairment. The common sequelae of ophthalmic involvement at birth are chorioretinitis, chorioretinal scars, macular scars, strabismus, cortical visual disturbances, nystagmus, and optic nerve atrophy [93]. Early diagnosis of chorioretinitis has been associated with the presence of other signs of CNS damage at birth. These data may suggest that early ophthalmologic evaluation is strongly recommended in neonates with suspected symptomatic congenital CMV infection [93]. Many factors contribute to the mortality and morbidity of congenital cytomegalovirus, including limited clinician and parental awareness of infection during pregnancy, inadequate maternal and newborn screening programs, and the absence of a licensed vaccine [94].

## 5. CMV Infection in Immunocompromised Hosts

CMV is one of the most common opportunistic pathogens in immunocompromised patients. In addition to congenital infection, CMV infection is a primary clinical concern in patients undergoing immunosuppressive therapy, such as solid organ and stem cell transplant recipients, cancer patients undergoing chemotherapy, and patients with AIDS [3,11]. Common manifestations of CMV disease in AIDS patients are retinitis, esophagitis, and colitis; other reported symptoms include encephalitis, neuropathy, and pneumonia [95]. In transplant recipients, CMV disease occurs in 11–72% of patients, especially during the first three months after transplantation when patients are maximally immunosuppressed [96,97].

Primary immunodeficiencies are a very diverse group of hereditary defects of the immune system [98]. Newborns with primary immunodeficiencies are also at significant risk for severe CMV infections, including pneumonia, interstitial pneumonia, hepatitis, neutropenia, thrombocytopenia, enteritis, retinitis, hemolytic anemia, and others [99,100,101].

AIDS-related CMV retinitis can cause vision loss and blindness. Mechanisms of blindness include destruction of the retina itself, retinal detachment, or uveitis (immune recovery uveitis [IRU]), which can occur with immune system reconstitution associated with well-tolerated hyperretroviral therapy [102,103]. Prior to the introduction of antiretroviral therapy (ART), it was estimated that approximately 40% of AIDS patients exhibited symptoms of CMV disease; however, with the introduction of ART, the incidence of these conditions in treated patients has decreased substantially [3,7,95,104,105,106].

## 6. CMV Retinitis

### 6.1. Epidemiology

CMV retinitis is a retinal infection of CMV in the retina after an initial or latent infection due to immunodeficiency in the host.

In the 1980s, CMV retinitis was reported as a major complication (approximately 30%) in AIDS patients [107,108,109,110,111]. CMV retinitis is likely to develop in AIDS patients when the number of CD4+ T-cells in the peripheral blood decreases below 50 cells/µL [103,112,113,114,115,116,117,118]. Notably, the introduction of anti-HIV therapy (i.e., ART) has increased CD4+ T-cell counts and reduced the proportion of the population with CD4+ T-cell counts below 50 cells/µL, thus reducing the incidence of CMV retinitis [102,103,117,119,120,121]; however, it has not yet been eradicated [117]. Therefore, CMV retinitis remains a serious clinical problem worldwide [24].

CMV retinitis is also reported in mildly immunocompromised patients with a variety of backgrounds, including stem cell transplantation for neoplastic diseases (e.g., malignant lymphoma and leukemia), chemotherapy for cancer patients, and immunosuppressive therapy for autoimmune diseases, diabetes, and aging. These conditions are referred to as chronic retinal necrosis [90,118,122,123,124,125,126,127]. The incidence of CMV retinitis has been reported to range from 0.2% to 5.6% in patients after hematopoietic stem cell transplantation or bone marrow transplantation [128,129,130].

### 6.2. Pathophysiology

The retina, a part of the posterior portion of the eye and an extension of the brain, possesses a unique characteristic that does not trigger the typical inflammatory immune response to antigen introduction [131,132,133]. This immune privilege is a significant factor in the development of diseases such as AIDS-related CMV retinitis. Loss of this immune privilege can result in inflammation and permanent vision-threatening damage, as is the case with AIDS-related CMV retinitis [24].

As previously discussed (see Section 2. The Mechanism of CMV Infection), the involvement of SOCS1 and SOCS3 in the signaling pathway during viral infection is well-documented [24]. These cytokines activate natural killer (NK) cells and T-cells and induce the death of virus-infected cells [134].

There are two different mouse models of experimental CMV retinitis: mice with corticosteroid-induced immunosuppression through systemic administration of corticosteroids and mice with MAIDS induced through systemic administration of a mixture of mouse-specific retroviruses called lymphoproliferative myeloid 5 [135,136]. Corticosteroid-induced immunosuppression in mice reduced the number of macrophages [137]; the total number and function of CD4+ and CD8+ T-cells [138,139,140]; and the expression, release, and function of inflammatory cytokines such as IFN-γ, TNF-α, and IL-2, leading to weakened immune responses and reduced inflammation [140]. SOCS1 and SOCS3 were not stimulated, and the severity of retinitis was reduced in the mouse model of experimental MCMV retinitis during corticosteroid-induced immunosuppression [141]. On the other hand, in the MAIDS mouse model, the number of macrophages and CD4+ and CD8+ T-cells increased in the late stage of infection [137]. In a mouse model of experimental MCMV retinitis in the late-stage MAIDS, SOCS1 and SOCS3 were abundantly produced by macrophages, granulocytes, microglia, and Müller cells, suggesting their contribution to the severity of retinitis [142]. These data suggest that SOCS1 and SOCS3 may be novel therapeutic targets for the management and prevention of AIDS-associated human CMV retinitis [24].

CMV retinitis is characterized by edema and necrosis of all retinal layers. Viral infection is observed in all layers of the retina, including retinal pigment epithelial cells, and infected cells are characterized by intranuclear inclusion bodies known as “owl’s eye cells”. The first target cells in the retina are the vascular endothelial cells, which hematogenously infect the inner retinal layers, inducing cell-to-cell transmission. There is a clear boundary between the diseased and healthy areas [143]. Infection is not usually observed in ocular tissues other than the retina, but it is rarely seen in the choroidal blood vessels or optic nerve [143].

### 6.3. Diagnosis

CMV retinitis is clinically diagnosed based on characteristic fundus lesions; however, as a more definitive criterion, the Standardization of Uveitis Nomenclature (SUN) Working Group in the United States established classification criteria for CMV retinitis in 2021. The classification criteria include (1) necrotizing retinitis with numerous small (<50 µm) satellite lesions; (2) immune compromise, either (a) systemic (e.g., AIDS, organ transplant, chemotherapy) or (b) ocular (e.g., intraocular steroid therapy or chemotherapy); (3) characteristic clinical features ([a or b or c] and d) including (a) wedge-shaped area of the retinitis, (b) hemorrhage appearance of the retinitis, (c) granular appearance of the retinitis, or (d) no or mild vitritis; (4) evidence of intraocular infection with CMV based on CMV-polymerase chain reaction (PCR) positive in aqueous humor or vitreous humor specimens. The diagnostic criteria require (1) and (2) and either (3) or (4). The exclusion criteria include (1) positive serology for syphilis using a treponemal test; (2) positive for HSV, VZV, or *Toxoplasma gondii* in intraocular fluid PCR (except in cases of immunocompromised patients, CMV infection, or CMV retinitis with characteristic clinical features, and the intraocular fluid has a positive PCR result for CMV) [143].

### 6.4. Clinical Findings

Although the clinical presentation of CMV retinitis in AIDS and other immunocompromised patients is similar and often unilateral at diagnosis, it eventually becomes bilateral. Subjective symptoms include decreased visual acuity, blurred vision, and visual field constriction; however, patients may be asymptomatic if the initial lesion is in the peripheral retina [1,90,122,123,124,126,144]. Therefore, active ophthalmologic screening based on CMV monitoring—not just symptoms—is necessary for early diagnosis of CMV retinitis [145].

Retinal lesions are clinically classified into the following three types [143,146]: (i) Indolent or granular variant: Small white granular exudate spots are observed in the peripheral area of the fundus that progress with fusion and enlargement. The central retina atrophies and turns greyish-white. It is rarely accompanied by retinal hemorrhage or vasculitis, and its progression is slow. (ii) Fulminant or hemorrhagic variant: Known to exhibit a “pizza pie” or “cottage cheese and ketchup” appearance, this variant is found in the posterior part of the fundus, characterized by thick white opaque lesions, edema, yellowish–white exudative plaques with retinal hemorrhage, and retinal vasculitis observed along the blood vessels. The progression is rapid, and the lesion expands in a wedge shape toward the periphery. Infiltration of the macula and optic nerve results in rapid loss of vision. The boundary between the affected and healthy areas is clear and characterized by white punctate lesions scattered on the healthy side of the active lesion. (iii) Perivascular retinitis: Retinal blood vessels, mainly large blood vessels, become dendritic and sheathed. This is a relatively rare variant observed in the early stages of disease onset that often coexists with other variants. Speculation regarding the etiology of frosted branch angiitis in AIDS includes an excess of CMV antigen with antigen–antibody complex deposition or direct cytomegalovirus infection of the vessel wall. Fine retinal dot deposits adjacent to the vein suggest antigen–antibody complex deposition [147].

As early treatment affects the visual prognosis of CMV retinitis, it is important to detect early lesions in the posterior part of the fundus. It is difficult to differentiate early lesions of CMV retinitis from those of HIV retinopathy, which is a retinal microcirculation disorder; however, the two can be easily differentiated using optical coherence tomography (OCT). OCT images show that, in CMV retinitis, the entire retinal layer structure has fallen off, and the retina is depressed. However, in HIV retinopathy, the lesions are localized to the inner retinal layer, the outer retinal layer is preserved, and the nerve fiber layer is elevated [148,149]. In obsolete CMV retinitis lesions, all of the layers of the retina are necrotic and thin with a lace-like appearance [150,151,152,153], and multiple retinal tears are easily formed when vitreous traction is applied, resulting in retinal detachment. Therefore, long-term follow-up is required even after the retinitis has subsided.

### 6.5. Treatment

CMV retinitis progresses relatively slowly, has fewer complications than acute retinal necrosis, and maintains relatively good visual function with the early detection of retinal lesions and appropriate antiviral therapy. However, if left untreated, exudative plaques can extend to the entire retina in about six months, leading to blindness; usually, the opposite eye also suffers vision loss within a year [3,103,116,154,155]. Therefore, if CMV retinitis is clinically suspected, antiviral drugs should be administered immediately.

The treatment of CMV retinitis is based on the systemic administration of ganciclovir, valganciclovir, foscarnet, or cidofovir, as well as the improvement of immune function [156,157,158]. As ganciclovir causes bone marrow suppression and foscarnet causes renal damage, the selection and dosage of the drug must be adjusted depending on the severity of the side effects. Given the systemic side effects, combined or single intravitreal administration of antiviral drugs can be selected. Initial induction therapy consists of the systemic or intravitreal administration of antiviral drugs for two to three weeks, followed by maintenance therapy until the retinitis is no longer active [159]. Sustained-release ganciclovir fixed in the pars plana of the ciliary body is sometimes administered as a vitreous injection; however, its production has been discontinued due to the decreasing incidence of CMV retinitis [143,160,161]. Intravitreal administration is not expected to be effective against CMV infections in the eye or other organs. In contrast, although retinal photocoagulation is considered ineffective for the prevention of retinal detachment, it is necessary to prevent retinal neovascularization and neovascular glaucoma in cases of peripheral retinal ischemia due to chronic retinal necrosis. Patients with retinal detachment require vitrectomy. Scleral buckling and silicone oil tamponade are often required to develop extensive retinal necrosis [162]. Factors that influence the visual prognosis include complications, such as retinal detachment, cataracts, optic neuritis, epiretinal membrane, and cystoid macular edema [163]. As anti-CMV drugs suppress viral replication in a virostatic fashion, dose reduction or discontinuation of the drugs can result in relapse of retinitis under persistent immunodeficient conditions. Therefore, it is necessary to improve immune function through the use of anti-CMV drugs, and treatment must often be continued for long periods of time [164]. In addition, immune recovery uveitis sometimes occurs as the immune function improves. This is thought to be an exacerbation of inflammation in the eye as an immune response to residual CMV antigens. The prevailing theory is that, when the CMV-specific T-cell response is restored through ART, it reacts with the CMV antigen present in the cells surrounding the CMV retinitis lesion, which has already subsided, and causes uveitis to manifest [146,165,166]. Therefore, long-term follow-up after CMV retinitis is essential.

## 7. CMV Corneal Endotheliitis

### 7.1. Epidemiology

Corneal endotheliitis causes specific inflammation in the corneal endothelium, resulting in irreversible corneal endothelial dysfunction as the disease progresses. Corneal endotheliitis is believed to be caused by infection, such as infection with herpes simplex virus (HSV). However, in 2006, Koizumi et al. reported corneal endotheliitis caused by CMV. As a result, it has become widely known that CMV infection results in corneal endotheliitis [167], and CMV corneal endotheliitis accounts for about 25% of all corneal endotheliitis cases [168]. This condition is more common in middle-aged men and can occur even in patients without systemic immune dysfunction. Indeed, it is frequently reported in Asian countries, including Japan and Singapore [18,19].

### 7.2. Pathophysiology

CMV corneal endotheliitis is thought to be caused by the reactivation of latent CMV in the corneal endothelium or neighboring tissues. Although the detailed pathophysiology remains unknown, immune responses are thought to be involved [19].

Zheng et al. suggested that anterior chamber-associated immune deflection (ACAID), a unique site of immune privilege [131,169,170], may play an essential role in the pathogenesis of herpetic corneal endotheliitis. In a rabbit model, they induced ACAID through inoculation with inactivated HSV followed by infection of the anterior chamber with live HSV. In this animal model, the findings of endothelial lesions were clinically very similar to human corneal endotheliitis. This result suggests that ACAID may be the underlying pathogenesis of herpetic corneal endotheliitis [171]. They speculated that, in CMV corneal endotheliitis, viral infection could also occur in the corneal endothelium if CMV was reactivated in the anterior chamber (including the corneal endothelium) and ACAID inhibited the control of viral growth through cell-mediated immunity [18,171].

As “owl’s eye cells” are commonly found in kidney, lung, and other organ specimens from patients with congenital or acquired CMV infection [2], it has been hypothesized that the “owl’s eye” morphology represents CMV-infected corneal endothelial cells [172,173]. However, as diagnostic corneal endothelial biopsies are invasive and are not commonly performed in patients with corneal endotheliitis, there is no direct evidence that CMV replicates efficiently in the corneal endothelial cells of patients. However, in vitro studies using primary cultured human corneal endothelial cells revealed “owl’s eye cells” found in infected corneal endothelial cells [174]. In addition, in patients with CMV corneal endotheliitis, “owl’s eye” morphological features—defined as large corneal endothelial cells with a highly reflective area at the nucleus surrounded by a low reflective halo—have been observed using in vivo laser confocal microscopy and specular microscopy [172,173].

The mechanism of CMV entry into the endothelial cells is the binding of the gH/gL/pUL128L PC to the cell receptors Nrp2 and olfactory receptor family 14 subfamily I member 1 to facilitate endocytosis of the viral particles into the endothelial cells. Subsequent gB activation by the gH/gL component (or gH/gL/gO) of the PC, followed by release of viral particles from the endosomal compartment into the cell, may be triggered by acidification of the vesicles [76,80,175]. Regarding the mechanism through which CMV causes specific inflammation in the corneal endothelium of individuals without immune dysfunction, the attenuation of anti-CMV-specific cytotoxic T-lymphocyte (CTL) activity has been reported; however, the specific details require further investigation [176]. In addition, as clinical observations have shown that corneal endothelial lesions always begin at the periphery of the cornea and spread to the center of the cornea, surrounding tissues such as the trabecular meshwork (TM) and ciliary body may serve as a reservoir for CMV [18,20] (discussed in detail in Section 8. CMV Iridocylitis). The discovery that bone marrow-derived cells migrate to the cornea [177] and the observation in nude mice that intraperitoneally inoculated MCMV reaches the cornea via the bloodstream [178] support this hypothesis [18].

### 7.3. Diagnosis

As CMV corneal endotheliitis is difficult to differentiate from HSV or varicella zoster virus (VZV) corneal endotheliitis based on clinical findings, viral detection using PCR is required. After corneal transplantation, it is necessary to differentiate it from rejection.

The Japan Corneal Endotheliitis Study Group has created diagnostic criteria based on PCR using aqueous humor and the clinical course that include the following: (I) positive PCR results for CMV DNA but negative results for HSV DNA and VZV DNA; (II) (i) corneal endotheliitis with coin-shaped lesions/linear posterior corneal precipitates (KPs) and (ii) local corneal edema with KPs plus two of the following findings (recurrent/chronic anterior uveitis, high intraocular pressure (IOP), secondary glaucoma, or corneal endothelial cell loss). It is recommended that (I) + (II) (i) be diagnosed as typical CMV corneal endotheliitis and (I) + (II) (ii) as atypical CMV corneal endotheliitis [19].

In addition, “owl’s eye cells” (macronuclear inclusion body) observed in the corneal epithelium using in vivo confocal microscopy can be useful for the diagnosis of CMV endotheliitis [172,179].

### 7.4. Clinical Findings

Corneal endotheliitis typically presents as localized corneal edema with KPs. In CMV corneal endotheliitis, small KPs arranged in an annular or oval shape (coin-shaped lesions) are observed in approximately 70% of cases [19]. In a typical case of CMV corneal endotheliitis, corneal edema starts in the peripheral region and progresses toward the center of the cornea. Linear KPs, such as the rejection line observed after corneal transplantation, are sometimes observed. Although most cases are unilateral, some are bilateral. There is no corneal infiltration or vascular invasion, and conjunctival hyperemia is often mild. Additionally, a decrease in corneal endothelial cell density (corneal endothelial dysfunction) is observed. Recurrent/chronic iridocyclitis, elevated IOP, secondary glaucoma, and cataracts may also accompany CMV corneal endotheliitis [180]. Vitreous opacity and inflammatory lesions are usually absent in the retina. As corneal endothelial dysfunction progresses, the entire cornea becomes edematous, and bullous keratopathy develops, resulting in a significant decrease in visual acuity [19].

To determine the effectiveness of treatment and the presence of recurrence of CMV corneal endotheliitis, measurement of the CMV DNA copy number in the aqueous humor using quantitative PCR (qPCR) is useful. It is also useful to observe the corneal endothelium using in vivo confocal microscopy, specular microscopy, or anterior segment spectral-domain optical coherence tomography (SD-OCT) to detect CMV corneal endotheliitis. In CMV corneal endotheliitis, “owl’s eye cells” are observed in the corneal endothelium using in vivo confocal microscopy or specular microscopy, and the number of these cells decreases upon successful treatment. Furthermore, in CMV corneal endotheliitis, SD-OCT images show hyperintensities of the corneal endothelium and protruding structures on the posterior corneal surface. As CMV corneal endotheliitis resolves with treatment, the intensity of the endothelial surface decreases, and the protruding structures disappear [173,179,181,182,183,184].

### 7.5. Treatment

Although there is no established treatment for CMV corneal endotheliitis, the effectiveness of combined treatment with anti-CMV drugs, such as ganciclovir, and steroids has been reported [19,185,186,187]. In mild or recurrent cases, only topical treatment with ganciclovir and steroids may be used; however, in cases of corneal endothelial cell loss or visual field impairment due to secondary glaucoma, the initial treatment may be combined with systemic administration. In cases of elevated IOP, topical glaucoma treatment and oral carbonic anhydrase inhibitors may be used as appropriate. It has recently been reported that ganciclovir gel, which has been approved for the treatment of HSV keratitis, is also effective in the treatment of CMV corneal endotheliitis. However, the number of cases reported was small, and long-term follow-up for recurrence is needed [188,189]. CMV corneal endotheliitis progresses relatively slowly, and corneal endothelial function can be maintained if treatment is initiated early. Careful follow-up and treatment are required to prevent recurrence. If clinical findings of recurrence (e.g., increased KPs or elevated IOP) are observed, anti-CMV treatment should be re-started or strengthened.

Corneal endothelial transplantation is required when corneal endothelial damage progresses to irreversible corneal edema (bullous keratopathy). Transplantation is desirable after negative results for CMV DNA in the aqueous humor are confirmed. After corneal endothelial transplantation, topical ganciclovir treatment should be continued when recurrence of CMV infection is observed [190].

## 8. CMV Iridocyclitis

### 8.1. Epidemiology

CMV can cause anterior uveitis (AU) and secondary glaucoma in immunocompetent individuals [20]. CMV iridocyclitis has been found in a large proportion of patients previously diagnosed with Posner–Schlossman syndrome or Fuchs heterochromic iridocyclitis [191,192]. Posner–Schlossman syndrome is a disease associated with transiently elevated IOP, iridocyclitis, and KPs [193], whereas Fuchs heterochromic iridocyclitis is a disease characterized by iris heterochromia, iridocyclitis, KPs, and cataracts as the main symptoms [194]. Chee et al. performed a PCR analysis of anterior uveitis with elevated IOP in 105 eyes and found that 24 eyes (22.9%) tested positive for CMV DNA. Among them, 18 eyes (75%) were clinically diagnosed with Posner–Schlossman syndrome, 5 eyes (20.8%) with Fuchs heterochromic iridocyclitis, and 1 eye with suspected herpetic iritis [185]. Most cases of CMV iridocyclitis have been reported in Asia—particularly in China and Japan—which may be attributed to the high rate of CMV seropositivity in Asia [20,195].

### 8.2. Pathophysiology

CMV-associated AU involves a complex immunoregulation; the most important immune responses to CMV are CD4+ and CD8+ T-cells. The immune response to CMV is significantly driven by CD8+ T-cells, which primarily target several viral proteins, including the immediate-early protein IE-1 and the tegument phosphoprotein pp65 [196]. Once the initial infection is controlled, highly activated effector T-cells, which secrete cytokines such as IFN-γ and kill virus-infected cells, initiate the apoptosis process [197]. CTLs and NK cells are important effector cells in the immune response to viruses. These killer cells induce cell death in infected target cells using a major mechanism, Fas-Fas ligand (FasL) and granule-mediated apoptosis [198,199]. The Fas/FasL system is involved in cell death induced by activation, and the granule exocytosis pathway utilizes perforin to transport granule enzymes (granzyme) to appropriate locations in target cells, where they cleave critical substrates to initiate DNA fragmentation and apoptosis [198].

Research conducted using MCMV models has confirmed that systemic CMV infection in an immunocompetent host triggers a severe inflammatory response and a latency period in the anterior part of the eye. Following systemic CMV infection, viral antigen staining has revealed that the virus is capable of invading and replicating in the iris, ciliary body, choroid, and cornea. This suggests that CMV infection leads to the infiltration and accumulation of antiviral CD8+ T-cells in the eye, leading to the development of tissue-resident memory T-cells. Even after the viral infection is controlled, foci of giant cells and viral cells persist in the iris sections, indicating the potential for CMV to cause latent infection in the eye [200,201].

Human iris cells are known to express CMV receptors such as integrin [202], PDGFα, and EGFR [203,204]. In addition, studies using human iris stromal cell culture models [204] have shown that CMV entry into human iris stromal cells regulated the actin cytoskeleton through the coordinated action of gB and heparan sulfate receptor, thereby influencing viral infectivity [205].

Usually, sera from immunocompetent patients with CMV iridocyclitis are positive for CMV IgG, but negative for IgM or CMV antigens. It has been suggested that CMV iridocyclitis is caused by the local reactivation of latent CMV [206]; however, the mechanisms that control CMV latency and reactivation remain unclear. It has been reported that surrounding tissues, such as the TM and ciliary body, may act as reservoirs for CMV [18,20]. In vitro experiments by Choi et al. revealed a homogeneous mass of electron-dense material and viral particles in the cytoplasm of TM cells, indicating that TM cells can effectively support CMV replication [207]. Histopathological studies of cadaveric eyes also showed that CMV inclusion bodies were localized mainly to the iris, ciliary body, and endothelial cells of the Schlemm’s canal [208].

Regarding the immune evasion of CMV AU, several studies have shown that immediate-early, early, and late-phase genes were stochastically transcribed during latent infection [209] and evaded immune responses by encoding multiple evasion proteins [210]. Latent CMV inhibits the antiviral activity of CD4+ T-lymphocytes by modulating CD14+ monocyte secretion, leading to the reactivation of CMV and a high recurrence rate of CMV iridocyclitis [117,201,211].

Regarding the cause of elevated IOP in CMV iridocyclitis, obstruction of the TM by inflammatory cells, proteins, residues, fibrin, or inflammatory precipitates may partially explain the initial increase in IOP. At the same time, substances suspended in the aqueous humor may increase its viscosity [212]. Furthermore, animal studies have shown that in the early stages of infection, the inflammatory edema that develops in the TM may obstruct the aqueous humor outflow tract [213]. Subsequently, permanent scarring of the TM and the Schlemm’s canal, as well as the formation of a vascular membrane in the anterior chamber angle, can block the flow of aqueous humor and lead to a permanent increase in IOP [214,215]. Steroids have also been shown to cause morphological and biochemical changes in TM, resulting in increased IOP [216]. The accumulation of inflammatory debris and cells in the angle has been noted to cause periapical anterior synechiae (PAS), which can lead to decreased outflow function and possibly increased IOP [216]. Inflammatory cytokines and chemokines may also have important functions in regulating CMV-induced outflow. Choi et al. have demonstrated the enhancement of transforming growth factor-β1 in CMV-infected TM cells in the early phase [207]. Stimulation of MCP-1 and IL-8 increases actin stress fiber formation, focal adhesion, and TM cell contraction, thereby potentially modulating the outflow and IOP [217].

### 8.3. Diagnosis

If the clinical findings described in Section 8.4 are observed in anterior segment uveitis, the presence of CMV DNA or antibodies should be determined in order to achieve a definitive diagnosis. CMV iridocyclitis is diagnosed through the detection of CMV DNA in the aqueous humor using PCR analysis or calculating the intraocular synthesis of CMV-specific antibodies in the aqueous humor using the Goldmann–Witmer coefficient [185,201,218,219].

### 8.4. Clinical Findings

According to a multicenter study in Japan [220], CMV iridocyclitis is characterized by (i) often unilateral, (ii) chronic or recurrent, (iii) small and coin-shaped KPs, (iv) an elevated IOP, (v) sometimes combined with corneal endotheliitis, and (vi) a decreased corneal endothelial cell density below 1000/mm^2^. Conjunctival hyperemia is mild and accompanied by symptoms including blurred vision and unilateral headaches [185,201]. KPs are characteristic, and when inflammation flares up, white (or transparent) small KPs are observed [221]. Diffuse and mild iris atrophy is observed, which can become severe in long-standing cases; iris heterochromia suspicious for Fuchs’ heterochromic iridocyclitis is sometimes observed [185,206]. In immunocompetent individuals, there are usually no vitreous opacity or retinal inflammatory lesions, and viral infections are limited to the anterior segment of the eye [222]. Secondary glaucoma due to repeated elevation of IOP, corneal endothelial cell loss (corneal endothelial deficiency), and concurrent cataracts can develop.

### 8.5. Treatment

Anti-CMV drugs are needed to manage CMV iridocyclitis; these include the topical administration of ganciclovir eye drops and the oral or intravenous administration of valganciclovir (a prodrug of ganciclovir with higher bioavailability [223]) or foscarnet, as well as anti-inflammatory drugs (e.g., topical steroids), pupil management drugs (e.g., mydriatic agents), and anti-high IOP treatments (e.g., glaucoma eye drops and oral carbonic anhydrase inhibitors) [201,219,224]. Systemic antiviral therapy is considered more effective than topical therapy in controlling inflammation [225]. The systemic administration of antiviral drugs requires attention to myelosuppression and renal damage [186], which should be monitored through regular blood tests. Although intravitreal ganciclovir has been found to have low levels of systemic toxicity, it has been reported that CMV DNA was not completely removed from the aqueous humor even after vitreous injection, suggesting that local/oral antiviral therapy should be continued after treatment with vitreous injection [201]. Treatment with ganciclovir gel has also been shown to be effective; however, comparative studies of its usefulness with systemic administration remain an issue [226,227]. Due to the high recurrence rate of CMV iridocyclitis, long-term treatment with antiviral drugs is recommended [219,222,224]. However, as long-term use of anti-CMV drugs may lead to CMV-resistant strains and systemic side effects, it is recommended that maintenance therapy should not exceed six months [201,228,229,230]. Elevated IOP due to chronic or recurrent iridocyclitis carries the risk of vision loss due to secondary glaucoma; therefore, the use of antiviral medication is necessary if CMV is present. However, if the combination of antiviral medication and topical glaucoma treatment is insufficient to reduce the IOP, filtration surgery may be required [185].

## 9. Conclusions

This paper provided a summary focused on the characteristics and treatment of retinitis, corneal endotheliitis, and iridocyclitis caused by CMV infection. In order to mitigate the infections caused by CMV in a wide range of organs, various clinical departments must work together to monitor the general condition of their patients, paying close attention to ocular symptoms.

## Data Availability

All data related to this work are presented and published here.
